# Dynamic characterization of water hammer in gangue fly ash slurry pipelines during valve closure

**DOI:** 10.1038/s41598-024-62504-2

**Published:** 2024-05-20

**Authors:** Yuxin Hao, Xuepeng Song, Chengshuai Wang, Bowen Fan, Kai Yang

**Affiliations:** 1 School of Energy and Mining Engineering, China University of Mining and Technology (Beijing), Beijing, 100083 China; 2 Shanxi Province Energy Vocational School (Shanxi Province Energy Staff Education Center), Taiyuan, 030012 China; 3 BGRIMM Technology Group, Beijing, 102628 China

**Keywords:** Gangue fly ash slurry, Water hammer pressure, Computational fluid dynamics, Transient flow, Energy science and technology, Engineering, Materials science

## Abstract

In the process of coal-filling mining, the gangue fly-ash slurry (GFS) needs to be transported over a long distance to reach the gobs. The abrupt closure of the valve during the transportation of GFS can result in a water hammer that significantly endangers the stability and safety of the pipeline transport system. To study the fluctuations in pressure induced by abrupt closure of the valve, experiments on the rheological parameters of gangue-coal ash slurry were conducted. Transient numerical simulations were carried out using the computational fluid dynamics method for various valve closing times. The results indicate that, with the increase of slurry concentration, the yield stress of the slurry significantly increases. When the concentration exceeds 76%, the increase in yield stress reaches 38.4% and 35.1%, respectively. Upon valve closure, the internal pressure of the slurry in the pipeline exhibits periodic dynamic oscillations. As the duration of valve closure increases, the frequency of periodic water hammer events decreases. The maximum water hammer pressure caused by valve closure decreases with the increasing distance between the valve and the closure point. At the same time, the intensity of maximum water hammer pressure fluctuations increases with the increase in slurry concentration and flow velocity in the pipeline. The results can provide references for water hammer protection and pipeline selection during the transportation of backfill slurry in mining.

## Introduction

Pipeline transportation has become the main method for transporting water, oil, chemicals, energy, and other resources due to its low cost, high safety, minimal pollution, and continuous and uninterrupted characteristics^1–4^. During the backfilling process in coal mining, the backfill slurry is extensively utilized by being transported through long-distance pipelines to fill the mined-out area of the coal mine. With mining depths reaching 1000 m, long-distance pipeline transport ensures the economical and efficient delivery of materials to the filling goaf^5^. During the transportation process in pipelines, sudden changes in the working status of pressure pipelines often cause significant pressure fluctuations, known as water hammer, which can lead to intense pipeline vibration and even rupture. Accurately understanding the generation, propagation, and attenuation laws of water hammer waves in solid–liquid two-phase flow is an important condition to ensure safe production and stable operation^6–8^.

The pressure variation law of slurry in transportation pipelines has always been a focus of basic research. Accurately assessing the pressure conditions of filling slurry inside the pipeline is crucial for safe operations and cost-effectiveness^9–11^. Research on the slurry used in coal mine filling revolves around the stable transportation process. Yang et al.^12^ studied backfill slurry flow in pipelines, simulating both straight and 90-degree elbow pipes. They found that bent pipes had higher local resistance loss and wider particle velocity distribution compared to straight pipes under similar transportation conditions. Wu et al.^13^ investigated the pipeline transportation characteristics of high-sliming paste from a copper mine in China by conducting tests on its rheological properties. Stable flow in pipeline transportation refers to a steady and consistent movement of slurry through the pipeline without sudden fluctuations or pressure surges. It typically occurs when the flow rate remains constant and there are no abrupt changes in velocity or pressure along the pipeline. On the other hand, water hammer is a transient phenomenon that occurs when there is a sudden change in the flow rate or velocity of the fluid within the pipeline. This change can be caused by various factors such as rapid valve closure, pump start/stop, or sudden changes in pipe geometry. Sarker et al.^14^ analyzed the efficacy of the power spectral density method for examining pressure waves resulting from water hammer, revealing insights into the fractal attributes and complex characteristics of unsteady flows. Urbanowicz et al.^15^ proposed a new solution for representing surface friction in water hammer simulations, emphasizing the importance of accurate modeling. Zhang^16^ conducted experiments to explore the pressure characteristics of direct water hammer in viscoelastic pipelines, revealing the mechanism of water hammer phenomenon within these pipeline systems. Kim et al.^17^ conducted simulations of water hammer induced by condensation in a steam pipeline, examining its features in both horizontal and vertical pipe segments. Utilizing Froude (Fr) and modified Jakob (Ja) numbers, they assessed water hammer strength and determined that smaller Fr and larger Ja are effective strategies for mitigating the pressure surge associated with condensation-induced water hammer. Behroozi et al.^18^ established a numerical model based on an implicit local multiple differentiation orthogonal method to simulate water hammer phenomena in pipeline systems consisting of a valve, pipeline, and surge tank. They found that the model's accuracy depends on the Courant number. Han et al.^19^ studied the impact of the number of closures and the closure pattern of ball valve pipelines on water hammer, offering guidance for safeguarding against water hammer incidents during the closure of ball valve pipelines. While investigating blockages and leaks during valve closure in pipeline systems, Fu et al.^20^ determined that the simultaneous closure of two valves efficiently mitigates pressure amplitudes within the pipeline, affirming the practicality of employing pressure waves generated through partially closed valves and the coordinated control of two valves to detect blockages and leaks. Warda et al.^21^ used the Finite Volume method to computationally simulate water hammer and column separation transient events in water pipeline systems to predict pressure fluctuations. Kandil et al.^22^ discovered that under identical operational conditions, materials with lower elasticity modulus were less susceptible to experiencing water hammer compared to those with higher elasticity modulus. Garg et al.^23^ analyzed the generation of water hammer pressure in two different pipeline materials, mild steel (MS) and glass-reinforced plastic (GRP), and compared it with the pressure in single-material pipelines. The results indicate that using a combination of GRP + MS pipelines can reduce transient pressure in water conductor systems. Yao^24^ employed multiscale asymptotic analysis to describe the attenuation of water hammer pressure waves caused by time-varying valve closures, establishing a simple empirical relationship between the attenuation of water hammer pressure waves and the predicted absolute mean period velocity of quasi-steady friction models. Research on pipeline water hammer is mostly concentrated in the field of water supply pipelines, with relatively fewer studies on water hammer in high-concentration slurries in coal mines.

**Figure 1:**
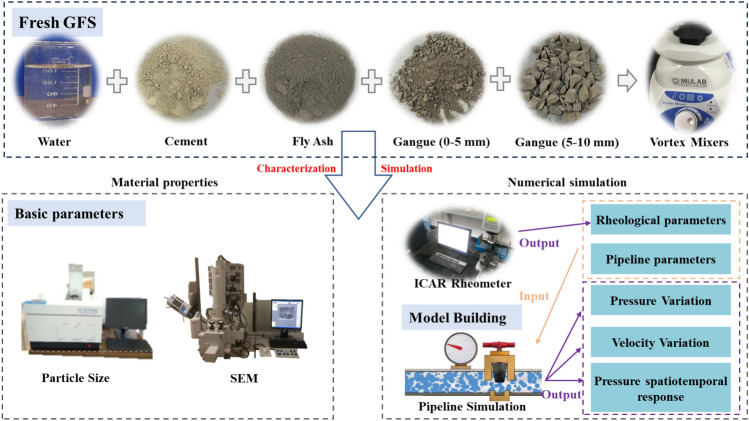
Technology roadmap.

Fresh GFS, denser than water and demonstrating compressibility, can experience substantial pressure variations with minor imbalances, thereby retaining significant energy. The surge in pressure resulting from water hammer often induces pipeline structural vibrations, subsequently altering the fluid flow pattern. The instantaneous pressure generated by water hammer can far exceed the normal operating pressure, resulting in significant destruction. Ensuring appropriate slurry concentrations is crucial for safer and more efficient pipeline transportation, safeguarding the stability of the filling slurry during transport. To illustrate this, we examine the backfill slurry pipeline of a mine as a case study. Figure 1 is the technical approach of this study, which involves investigating the rheological properties of GFS and its relationship with slurry concentration through laboratory rheological experiments. Due to the difficulty in detecting the transient behavior of slurry inside the laboratory pipeline during simulated valve closures, numerical simulation methods were employed to investigate the pressure changes of GFS inside the pipeline when the valve is suddenly closed, utilizing the Eulerian multiphase method to simulate water hammer situations at different velocities for GFS within the pipeline. The research findings aim to guide the determination of the flow status of coarse particle filling slurry and predict water hammer pressure.

## Experiments

### Materials

**Figure 2:**
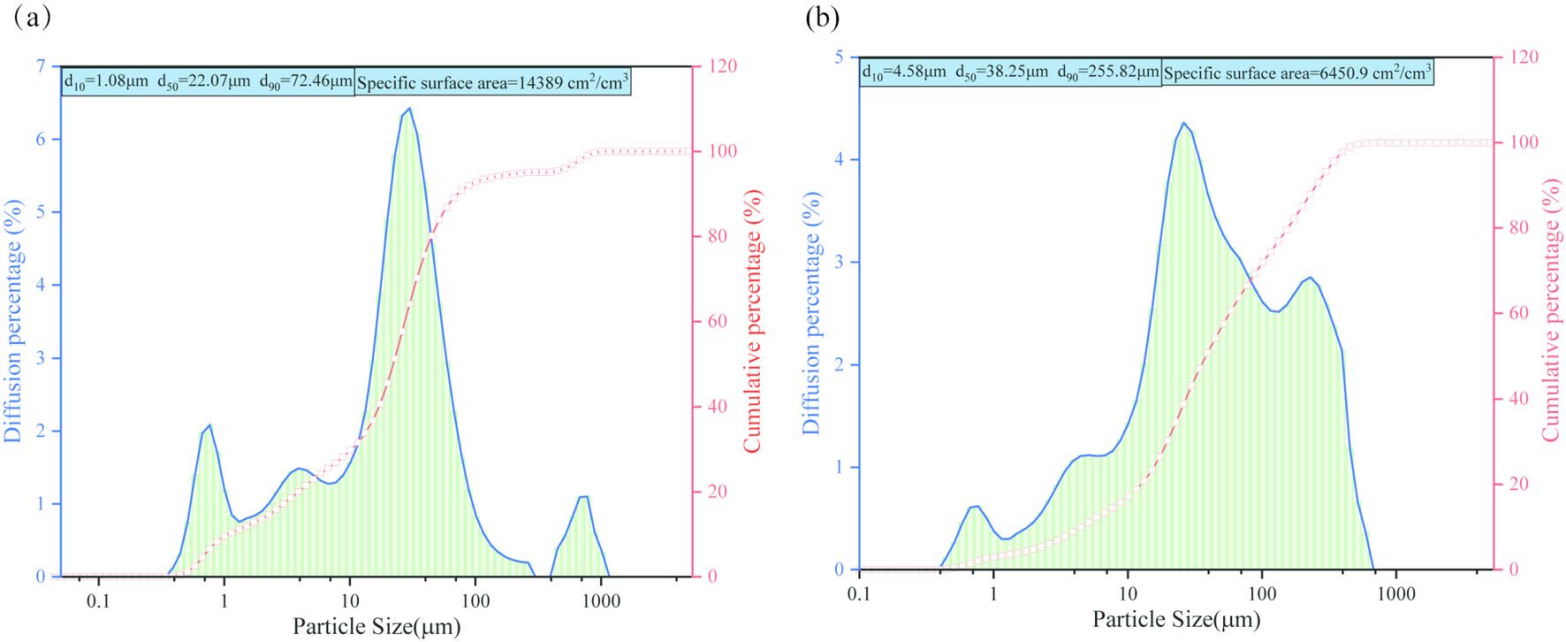
Particle size distribution of (**a**) cement (**b**) fly ash.

**Figure 3:**
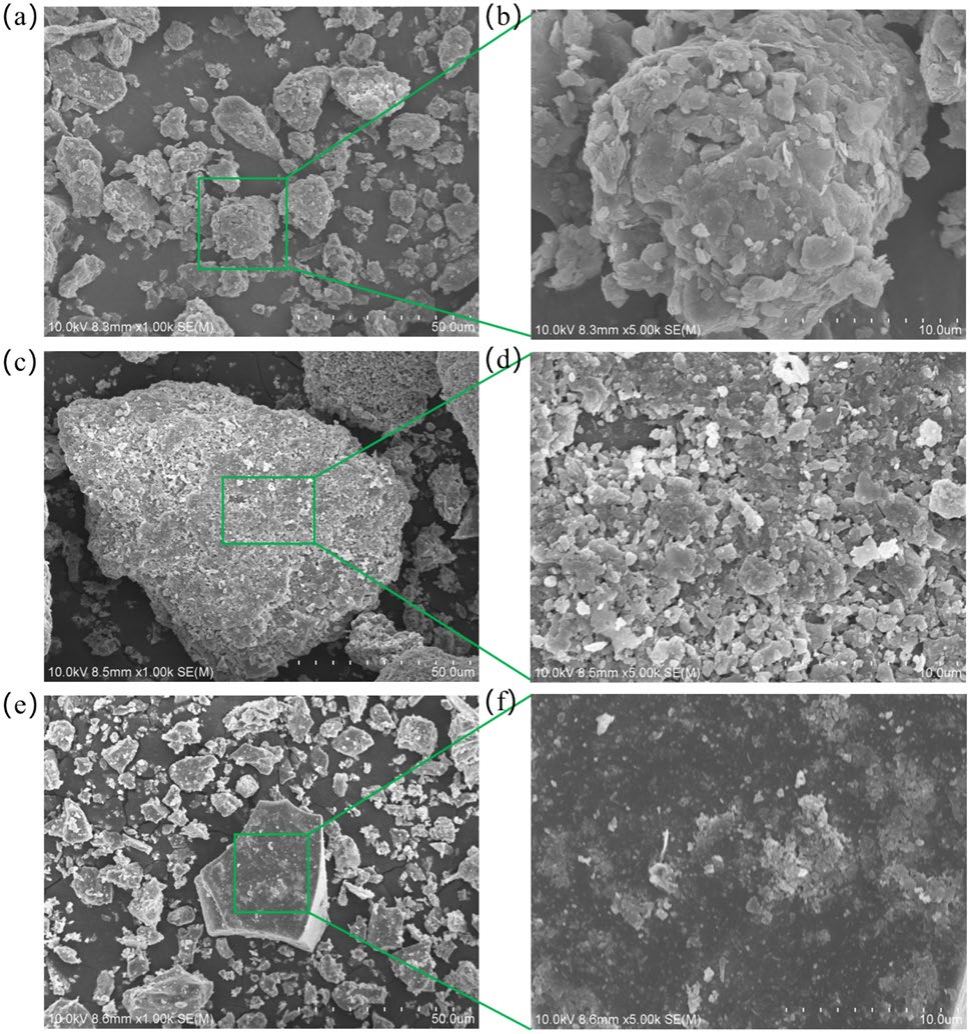
Microstructure of the gangue (**ab**) and fly ash (**cd**) and (**ef**) cement.

The materials used in this study include gangue from a coal mine, fly ash from a nearby power plant, and ordinary Portland cement. The fundamental physical properties of gangue (water absorption, moisture content, apparent density, particle size distribution) are detailed in the reference^25^. Figure 2 illustrates the particle size distribution of fly ash and cement. The microscopic structures of coal gangue, fly ash, and cement are shown in Fig. 3. Cement exhibits irregular granular particles, while fly ash appears as quasi-spherical particles on the surface. The smaller average particle size of fly ash compared to cement enables it to fill the gaps between cement particles, thereby improving the flowability of the slurry during pumping and lubrication of the pipeline.

### Rheological test

The specific proportions of the slurry used in the experiment are as detailed in Table 1. Rheometers were utilized to measure the rheological properties of the slurry. The Bingham model is used to describe the rheological properties of GFS, represented by the equation^26,27^:

1 $$\tau = \tau_{0} + k\gamma$$

where τ, τ_0_, *k*, and γ are shear stress (Pa), yield stress (Pa), plastic viscosity (Pa·s) and shear rate (1/s), respectively.

**Table 1:** Mix proportions design.

Gangue/g	Fly ash/g	Cement/g	Water/g	Concentration/%
322.8	80.8	40.4	156	74
327.3	81.8	40.9	150	75
331.6	82.9	41.5	144	76
336	84	42	138	77
340.4	85.1	42.5	132	78

**Figure 4:**
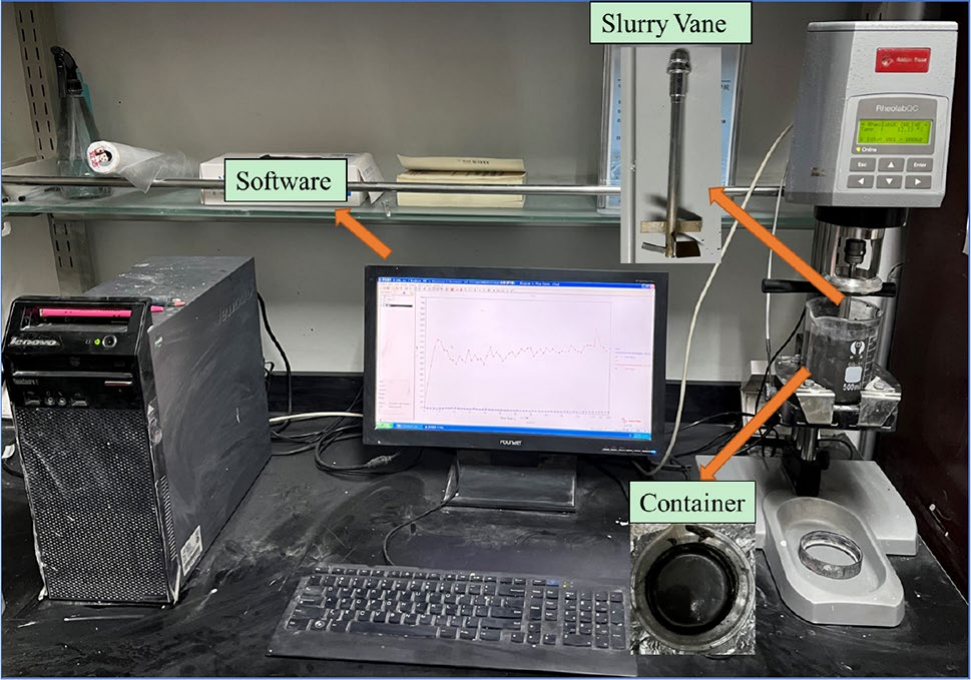
The rheological testing device.

The rheological test device, depicted in Fig. 4, is the RheolabQC rotary rheometer. With a maximum allowable particle size of 40 mm, the rheometer aligns with the testing requirements for the materials utilized in this study. The experimental procedure involves the following steps:

(1) Configure the rheometer's test mode to CSR (Control Shear Rate) mode, adjusting the shear rate within the range of 1 to 120 1/s. (2) Prepare GFS based on the mixing proportions outlined in Table 1, pour the mixed material into the container, and insert the blades into the mixture. The test temperature is controlled at 23°. (3) Initiate the rheological curve testing by clicking the start button. Record the torque at different speeds, and the rheological parameters are automatically calculated.

## Simulation

### Method

The existing methodology for analyzing water hammer flows in pipelines is based on certain assumptions: The flow is compressible and experiences elastic deformation when subjected to high pressures, with negligible relative changes in density. The speed of the pressure water hammer wave is considerably higher than the velocity of the liquid flow. The instability events induced by water hammer during pipeline transport pose a potential threat to the safety processes within the system and are the most challenging aspect in the design of such systems. The method of characteristics (MOC) utilized in water hammer calculations has a broad basis of application. It primarily comprises continuity equation and momentum equation^20,22^:

2 $$g\frac{\partial H}{{\partial {\text{x}}}} + V\frac{\partial V}{{\partial {\text{x}}}} + \frac{\partial V}{{\partial {\text{t}}}} + f = 0$$

3 $$V\frac{\partial H}{{\partial {\text{x}}}} + \frac{\partial H}{{\partial t}} + \frac{{a^{2} }}{g}\frac{\partial V}{{\partial {\text{x}}}} = 0$$

where *g*, *H*, *V*, *x*,* t*, *f* and* a* are gravitational acceleration (m/s^2^), pressure (m), flow rate (m/s), pipe length variable (m), time variable(s), friction coefficient and wave speed (m/s), respectively.

In Eq. (2), *f* represents friction loss, which is the aggregate of both constant and non-constant components, combining to influence the overall efficiency and performance of fluid flow. The expression is depicted as follows^20^:

4 $$f = \frac{{f_{r} }}{2D}V|V| + \kappa \left( {\frac{\partial V}{{\partial t}} - a\frac{\partial V}{{\partial x}}} \right)$$

where $f_{r}$ and κ are hydraulic friction coefficient and Brunone coefficient of friction, respectively. The semiempirical and semitheoretical formula for κ is

5 $$\kappa = \frac{{\sqrt {C^{ * } } }}{2},\quad C^{ * } = \frac{7.41}{{Re^{m} }},\quad m = \log \left( {\frac{14.3}{{Re^{0.05} }}} \right)$$

*a* can be calculated from Eq. (6):

6 $$a = \sqrt {\frac{{\frac{{E_{v} }}{{\rho_{v} }}}}{{1 + \left( {\frac{{E_{v} }}{{E_{s} }} - 1} \right)C_{v} + \frac{{E_{v} D}}{{E_{p} \delta }}}}}$$

*E*_*v*_, *E*_*s*_, *E*_*p*_ are the volume compression elastic modulus of the slurry, the elastic modulus of solid particles, and the elastic modulus of the pipe (Pa), respectively. *ρ*_*v*_ and *C*_*v*_ representing the slurry density (g/cm^3^) and volume concentration (%). δ and D represent the thickness of pipe(mm) and the inner diameter of the pipeline (mm).

The formula for determining the elastic modulus of GFS is:

7 $$E_{v} { = }\frac{{E_{w} }}{{1 - C_{v} }}$$

where *E*_*v*_, *E*_*w*_, *C*_*v*_ are the elastic modulus of GFS slurry, the elastic modulus of water(2.1 × 10^9^ Pa) and the volume concentration of mineral slurry, respectively.

**Figure 5:**
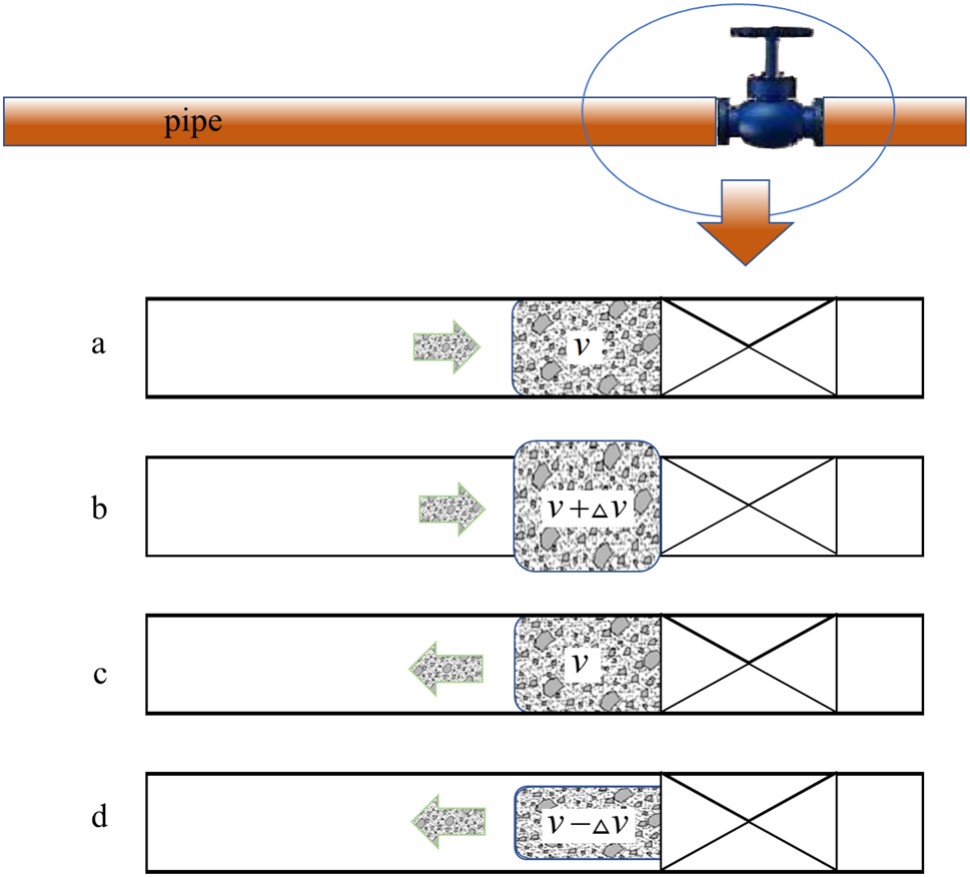
Water hammer cycle schematic.

As shown in Fig. 5, during the flow process, the sudden closure of the valve causes the slurry to compress and expand in the ab phase. After reaching a certain energy accumulation, in the cd phase, it reverses flow direction, resulting in reduced slurry density. This repeated cycle leads to a rapid change in the speed of the slurry, ultimately resulting in the occurrence of water hammer phenomenon. A pressure fluctuation zone is formed near the valve. To facilitate a better comparison of hydraulic transient pressure in the subsequent context, the Allievi–Joukowsky formula^28^ is introduced to represent the pressure surge during water hammer events in pipelines:

8 $$\Delta P_{{}} = \frac{{aV_{0} }}{g}$$

where* a* and* V*_*0*_, are the wave speed and the steady-state velocity.

**Figure 6:**
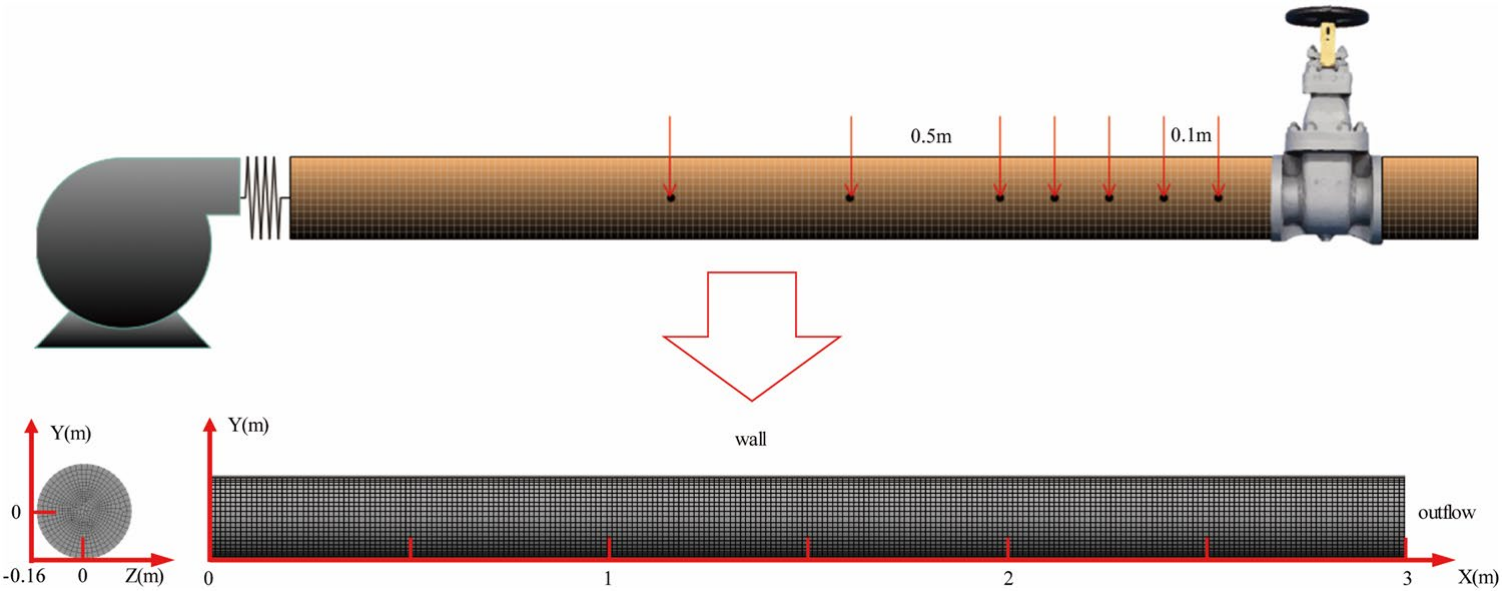
The size and grid of the computational pipe model.

The velocity of conveying high-concentration slurry in the pipeline should be lower than that of water transport. The diameter of solid particles in GFS is much smaller than the pipeline radius, hence the slurry can be considered a pseudo-homogeneous flow^11^. Typically, the reflection source is negative under ordinary circumstances, and under similar conditions, the indirect water hammer pressure is less than the direct water hammer pressure. Numerical methods were used to simulate the transient experimental scenario when the slurry valve is suddenly closed. The experimental setup comprises a pressurized pump-pipeline-valve system, with the transient event induced by the abrupt closure of the downstream valve. The model established in consideration of the actual situation of mine filling pipelines is shown in Fig. 6:

Generally, water hammer resulting from valve closure tends to inflict primary damage on the pipe region in close proximity to the valve. In this study, we have chosen the area located three meters ahead of the valve as our research focus. Within this selected zone, pressure and velocity parameters are assigned for detailed analysis. The pipeline, featuring a diameter of 160 mm, is equipped with a filling pump on the left side to provide initial pressure for the slurry within, while the valve is situated on the right side. To ensure a comprehensive assessment, monitoring points are strategically placed along the pipeline. Specifically, within the first 0.5 m near the valve core entrance, monitoring points are established at 0.1 m intervals. Beyond this initial 0.5 m distance from the valve core, monitoring points are positioned at 0.5 m intervals. These monitoring points enable the observation of pressure variations at each designated location over time. Concurrently, a thorough analysis of the fluid flow state within the pipeline is conducted. It is noteworthy that the typical speed range for slurry transport falls within 0.8 to 2 m/s. Considering the actual conditions in actual mine conditions, we have set the slurry speed within the range of 1 to 1.8 m/s to accurately simulate pipeline pressure transport. Water hammer is a transient fluid dynamics phenomenon that typically occurs at the moment of valve closure. Opting for a narrower closing time range serves to mitigate the computational complexity associated with numerical simulations, thereby enhancing overall simulation efficiency. This, in turn, accentuates the fluctuations in water hammer pressure. To effectively capture the impact of the water hammer phenomenon within this brief yet pivotal time frame, the cutoff flow time is set between 0.001 and 0.1 s, with the related parameters outlined in Table 2.

**Table 2:** Simulation parameters.

Parameters	Value
Density (kg/m^3^)	1900
Viscosity	1.5–2.6
Concentration (%)	74–78
Velocity-inlet (m/s)	1–1.8
Time step(s)	1 × 10^–3^

### Grid independence verification

To improve computational speed and stability, recreate the non-steady-state motion encountered by the slurry during regular flow. Begin with steady-state calculations with the valve fully open. Once the flow stabilizes, utilize the converged solution as the initial value for non-steady-state calculations. Validate using the steady flow velocity attained within the pipeline and conduct comparative simulations on pressure drops in different meshed pipelines.

**Figure 7:**
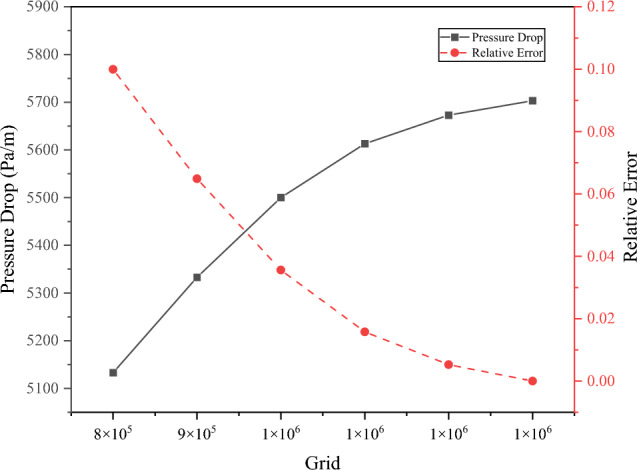
Comparison results of mesh calculations.

The computational domain utilized a combination of hexahedral structured grids and tetrahedral unstructured grid techniques. The grid count spanned a spectrum from fewer to more, representing varying levels of discretization in the computational domain. During numerical simulations, the valve was operated in a fully open position, and consistent inlet and outlet boundary conditions were applied. The primary objective of these simulations was to calculate the average pressure drop within the pipeline. The computational results for each scheme are shown in Fig. 7. Grid independence verification was performed by analyzing the influence of grid quantity on pipeline pressure drop. The pipeline pressure drop increased with an increase in the number of grids. When the grid increased to one million, the relative error in pressure drop change was below 5%, indicating that this scheme met the requirement of grid independence. As the number of elements increased, the unit pressure drop tended to converge. Increasing the grid elements continuously reduced the gaps and provided more accurate results. However, the improvement in accuracy was very limited. To maintain a balance between computational efficiency and accuracy, scheme 3 was chosen for subsequent simulations.

### Boundary conditions

The dynamic numerical simulation of the valve's closure is executed through transient calculations, considering various closing durations. The closure mechanism of the valve is achieved through the application of dynamic mesh methods. The initial values for transient numerical calculations are obtained from the steady-state numerical calculations of the fully open valve mentioned earlier^19,29,30^. Defining the slurry as a compressible liquid, the inlet and outlet boundary conditions were established as pressure-fixed. Based on experimental data, the inlet featured a total pressure of 4 × 10^5^ Pa, while the outlet's static pressure was set at 0 Pa, with a reference pressure of 101,325 Pa. Employing the two-equation SST k-w model for turbulence, a time step of 10^–3^ s was chosen to adhere to the Courant criterion^31^. The overall calculation time was contingent upon the closing times, allowing for a comprehensive study of transient dynamics. This approach ensures a detailed examination of the slurry's behavior during closure, maintaining accuracy while streamlining the presentation of key parameters and methods. The overall calculation time was contingent upon the closing times, allowing for a comprehensive study of transient dynamics. This approach ensures a detailed examination of the slurry's behavior during closure, maintaining accuracy while streamlining the presentation of key parameters and methods.

## Results and discussion

### Rheological experiment results

**Figure 8:**
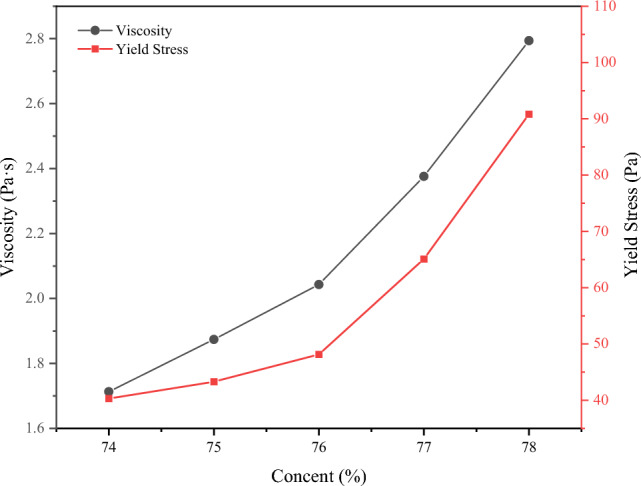
Rheological parameters of GFS materials at different concentrations.

The experimental results depicted in Fig. 8 illustrate a substantial impact of slurry concentration on rheological parameters. With an increase in concentration, the material's flow behavior exhibits a clear nonlinear trend. Notably, both viscosity and yield stress experience significant increments with concentration, showing a respective increase of 38.4% and 35.1% after reaching a concentration of 76%. Additionally, viscosity displays a corresponding significant increase after reaching the 76% concentration. The higher yield stress demands more energy consumption to initiate slurry flow within the pipeline, signifying a notable increase in flow initiation resistance at higher concentrations. Concurrently, the higher viscosity associated with higher concentrations leads to greater pressure drops during slurry flow. These findings can provide a theoretical basis for setting simulation parameters in subsequent slurry transport.

### Temporal evolution of the water hammer pressure law

**Figure 9:**
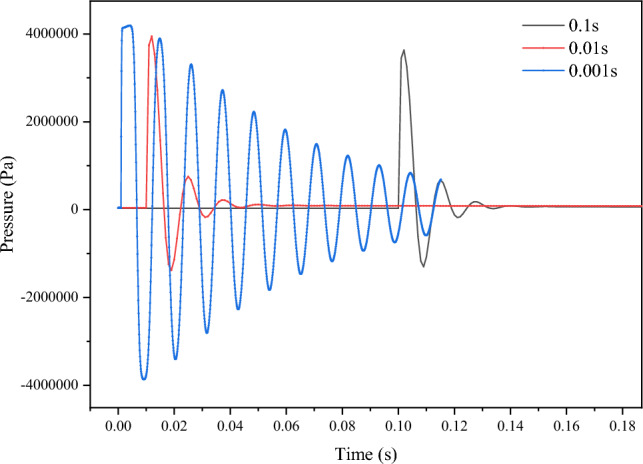
Variation of pressure with time at the point 0.1 m from the valve.

Figure 9 illustrates pressure variations observed at a monitoring point located 0.1 m from the valve. The axial pressure within the pipeline exhibits periodic oscillations over time, characterized by a certain amplitude of fluctuating attenuation. It is noteworthy that the pressure fluctuations show a trend of shorter cycles in the early stage and longer cycles later in the time series. The occurrence of negative pressure values stems from the detection of axial stress at the monitored point within the simulation process. As the slurry continuously reflects and undergoes compression, negative axial stress manifests at that point. Notably, when the closing time is 0.001 s, the resulting pressure fluctuations are more intense, featuring shorter periods of oscillations. However, with extended closing times (0.01 s and 0.1 s), the amplitude of the pressure curve fluctuations significantly diminishes. The initial pressure rise in fluctuations stems from the abrupt valve closure, causing the fluid ahead to cease flowing while the rear slurry continues to exert pressure on the pipeline. Subsequent reflections lead to further pressure decrease and reach their maximum in the reverse direction. The substantial and prolonged initial pressure rise at the valve end is attributed to the earlier rapid flow velocity of the slurry, characterized by greater inertia.

### Distance-dependent water hammer pressure

**Figure 10:**
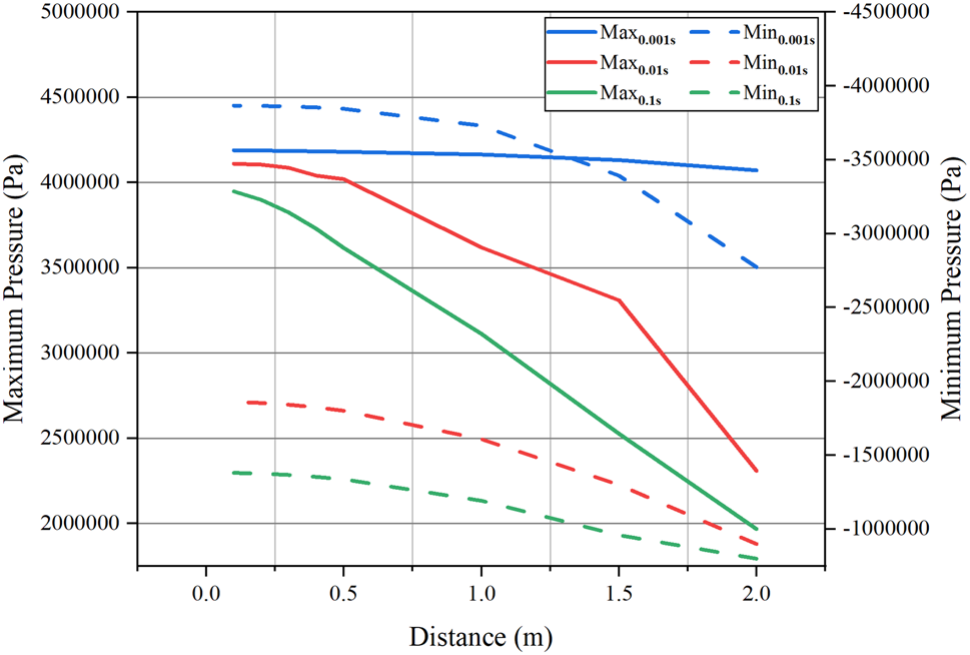
Relationship between maximum pressure and valve distance.

Figure 10 depicts the maximum positive pressure at various points, revealing that the maximum pressure within 0.5 m of the valve closing point is relatively consistent. A shorter valve closing time corresponds to higher maximum pressure, resulting in a more pronounced impact on the pipeline. With increasing distance, there is a decreasing trend in the maximum pressure. At a valve closure time of 0.001 s, the rate of decrease in maximum pressure is comparatively lower, indicating a minor downward trend. Extending the valve closure time leads to a more significant decrease in maximum positive pressure, showing an almost linear declining trend when closed at 0.1 s, signifying substantial energy loss. Simultaneously, an extremely short closure time of 0.001 s results in much higher water hammer positive pressure compared to 0.01 s and 0.1 s. Conversely, the water hammer negative pressure is much smaller than the positive pressure. Concerning the variation trend, the pressure change rate induced by closing the valve at 0.001 s is relatively smooth, suggesting a lower impact of distance on the maximum water hammer pressure and faster propagation of the water hammer wave. The maximum water hammer pressure decreases with increasing distance from the valve end due to energy loss during pressure wave propagation, caused by friction between the slurry and the pipeline wall. Simultaneously, repeated changes in fluid movement direction within the slurry result in energy loss, with greater inertia leading to increased energy loss over longer distances.

### Spatio-temporal response to water hammer with different concentrations

**Figure 11:**
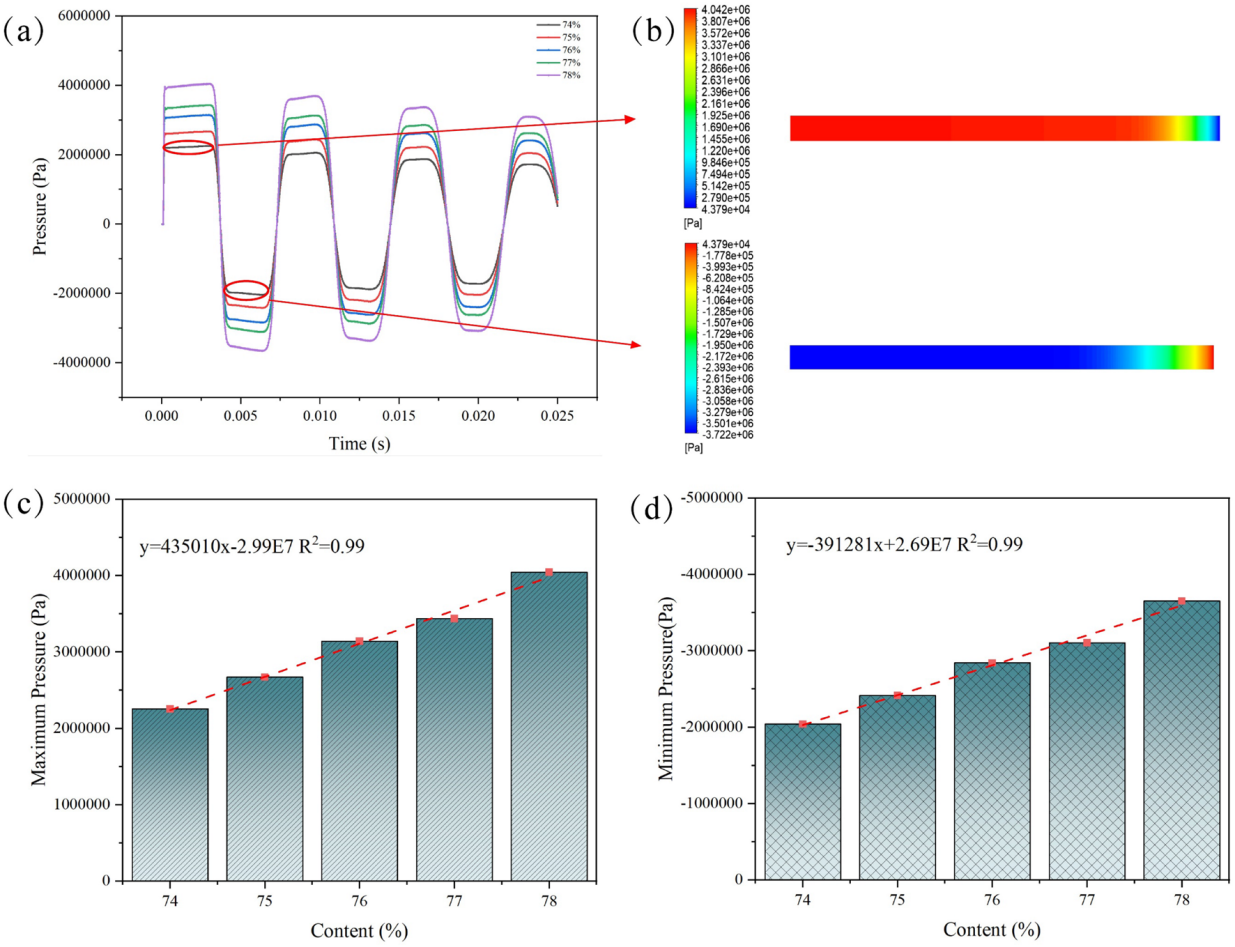
Variation of pressure for different concentrations (**a**) 74–78% concentration (**b**) 74% concentration GFS max–min pressure contours (**c**) max water hammer pressure (**d**) min water hammer pressure.

Observing the trends in water hammer responses across various slurry concentrations reveals a consistent fundamental behavior (Fig. 11a). However, a detailed examination of the spatiotemporal responses under different solid particle concentrations unveils distinctions in achieving maximum positive pressure. Specifically, the maximum positive pressure demonstrates variable changes with an increase in solid particle content, showing a 16% increase at a concentration of 78% compared to 74%. Further analysis indicates relative growth rates ranging from 2.1 to 4.5% during the transition from 74 to 78% concentration. Meanwhile, the duration of maximum pressure remains relatively constant across different concentrations. This phenomenon arises from the augmented quantity of solid particles at higher slurry concentrations, increasing the likelihood of these particles altering their motion states during water hammer events. Consequently, more kinetic energy is released, resulting in an elevation of the maximum pressure level. Taking the water hammer phenomenon at 74% concentration as an example (Fig. 11b), pressure fluctuations induced by valve closure exhibit gradual propagation within the pipeline. Through the observation of pressure cloud charts depicting water hammer positive and negative pressures, it becomes evident that at the moment of valve closure, the generated pressure disturbance rapidly propagates. The maximum water hammer pressure observed at a distance of 0.1 m from the valve exhibits a discernible upward trend with increasing concentration (Fig. 11c, d). It can be seen that the growth rate of the maximum positive pressure is greater than that of the negative pressure. At the same time, the extreme value of water hammer pressure is positively correlated with the increase of concentration. This suggests that the heightened concentration may induce alterations in the GFS's viscosity, thereby initiating the water hammer phenomenon and resulting in an elevated water hammer pressure at the respective location.

### Spatio temporal response to water hammer with different velocities

**Figure 12:**
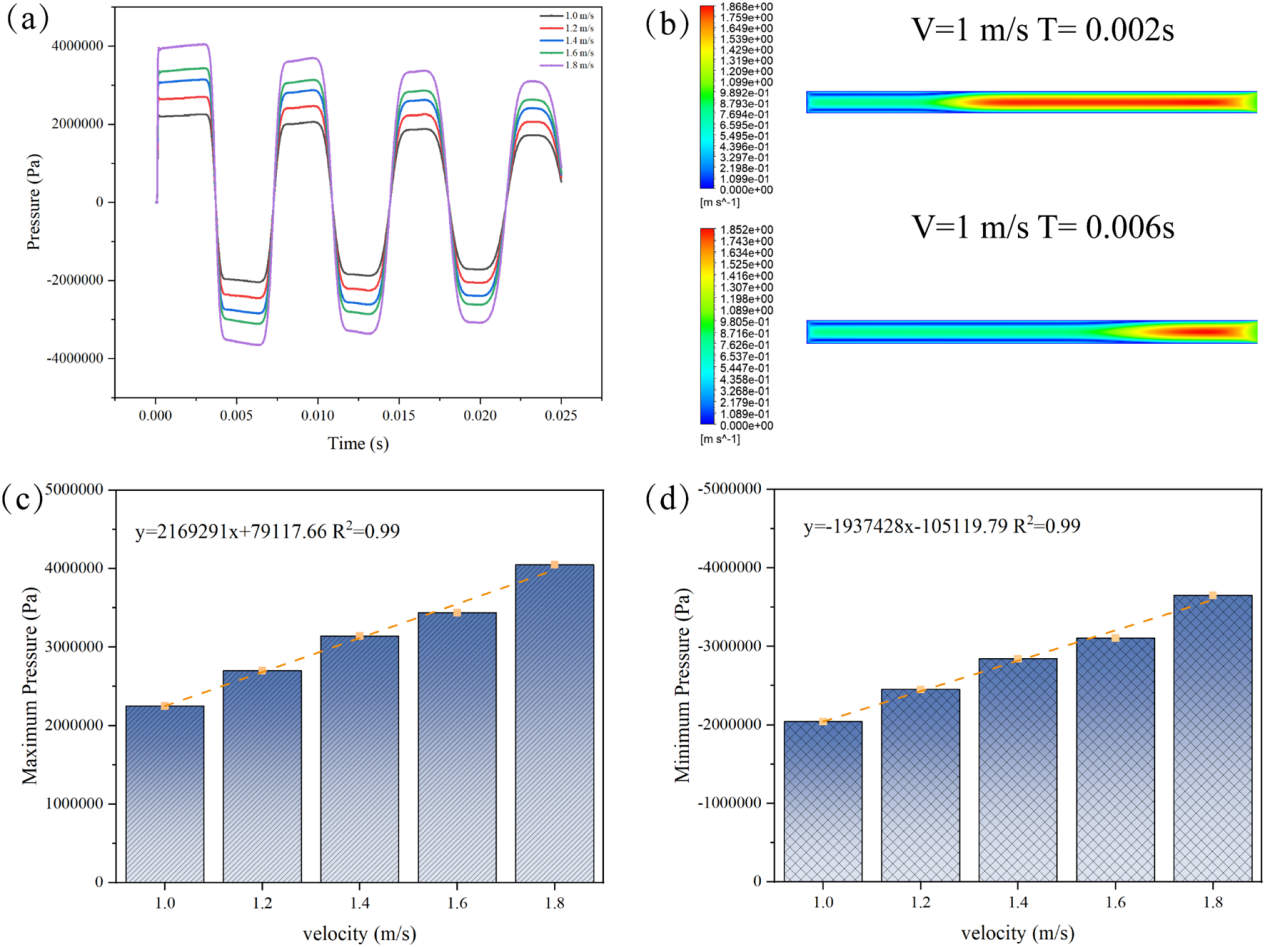
Variation of pressure for different velocities (**a**) 1–1.8 m/s (**b**) 1 m/s GFS max–min pressure contours (**c**) max water hammer pressure (**d**) min water hammer pressure.

Figure 12a indicates that, during the monitoring of water hammer phenomena at different flow velocities, the maximum water hammer pressure exhibited a positive correlation with increased velocity when maintaining a constant flow speed. Specifically, a rise in velocity from 1 to 1.8 m/s resulted in a significant 79.45% increase in the maximum positive pressure. Further subdivision of velocity increments of 0.2 m/s revealed a gradual reduction in pressure increments, decreasing from 22.18 to 11.07%. The maximum negative pressure at 1.8 m/s increased by 79% compared to 1 m/s, displaying a trend similar to that of the positive pressure. Within each 0.2 m/s increment in velocity, the increments in negative pressure were successively 16.4%, 14.0%, and 12.64%, indicating a significant increase despite the gradual reduction in increments. With the rise in the flow rate, the energy released by the pressure gradually increases, resulting in a progressively larger rise in the maximum water hammer pressure. Analyzing the velocity cloud map (using 1 m/s as an example), as shown in Fig. 12b, revealed that at the peak water hammer pressure, the maximum velocity was associated with the flow core inside the pipeline. The velocity exhibited the characteristic of the central flow core occupying a relatively large space within the pipeline. At this moment, the energy within the pipeline fluid was higher, resulting in a relatively substantial impact on the valve. However, at 0.006 s, the maximum velocity appeared near the valve. The kinetic energy of the slurry inside the pipeline decreased, and its fluctuation in flow velocity was identified as the fundamental cause of pressure wave fluctuation. The elevation in flow rate results in an increase in kinetic energy (Fig. 12c, d). As can be seen, with the increase of flow velocity, the extreme value of water hammer pressure shows an upward trend, and the slope of the curve in Fig. 12 c is greater than that in d, indicating that the rate of the upward trend of water hammer positive pressure is greater. When the valve is closed, the liquid's kinetic energy cannot dissipate instantly, and a portion of it is transformed into the water hammer effect, consequently causing an upsurge in the maximum water hammer pressure detected at a distance of 0.1 m from the valve. Consequently, the escalation in slurry flow rate indirectly contributes to a heightened maximum water hammer pressure due to the associated increase in kinetic energy.

## Conclusion

This study focuses on the water hammer evolution patterns caused by the sudden closure of valves in the pipeline transporting GFS. Following a thorough analysis of the experimental raw material properties, we successfully determined the rheological parameters of high-concentration slurries. Our investigation delved into the pressure variation patterns exhibited when slurries of different concentrations and velocities encounter sudden valve closure in pipelines. The main research conclusions are as follows:

1. Slurry rheological changes: With an increase in slurry concentration, the material's fluidity decreases. This is accompanied by a notable rise in the yield stress of the slurry. Beyond a concentration of 76%, the yield stress increment reaches 38.4% and 35.1%, respectively. The elevated yield stress leads to heightened energy consumption during the initiation of slurry flow, while increased viscosity contributes to greater energy consumption throughout the slurry's flow process.
2. Pressure oscillations: The pressure inside the pipe displays periodic oscillations at various time intervals. The substantial initial pressure surge induced by the 0.001 s valve closure is significant and persists for an extended period. Fluctuation cycles are characterized by relatively shorter durations in the early stage and longer durations in the later phase.
3. Distance impact on water hammer: The maximum water hammer pressure within the pipeline diminishes with an increase in distance from the valve. Shorter valve closure times correspond to a more pronounced decreasing trend in the maximum water hammer pressure.
4. Concentration and velocity effects: The relative growth rates of maximum positive pressure during the transition from 74 to 78% concentration range from 2.1 to 4.5%. At a constant flow velocity, the maximum water hammer pressure experienced by the slurry increases with higher flow velocities. The maximum positive pressure at 1.8 m/s is significantly greater than at 1 m/s, with an increase of 79.45%. With each increment of 0.2 m/s, the pressure increase gradually reduces from 22.18 to 11.07%.

## Data Availability

Data associated with this research will be made available from the corresponding author on reasonable request.
